# Aromatherapy was used to explore the sedative and hypnotic effects of *Moringa seed* essential oil on insomnia rats

**DOI:** 10.1002/fsn3.4484

**Published:** 2024-11-12

**Authors:** Shaofeng Wei, Ruijie Chen, Xiaoyi Liu, Haoran Ma, Yang Peng, Xiefei Wu, Yong An, Xinru Wang, Peng Luo

**Affiliations:** ^1^ The Key Laboratory of Environmental Pollution Monitoring and Disease Control, Ministry of Education, Guizhou Provincial Engineering Research Center of Ecological Food Innovation, Collaborative Innovation Center for Prevention and Control of Endemic and Ethnic Regional Diseases Co‐constructed by the Province and Ministry, School of Public Health Guizhou Medical University Guiyang Guian New Area China

**Keywords:** aromatherapy, hypnotic effects, *Moringa seed* essential oil, sedative

## Abstract

*Moringa* is a type of plant that is used both for medicinal and food. *Moringa seed* (MS) are rich in volatile oil and have initially been employed to treat diseases of the nervous system. Insomnia, a prevalent neurological disorder, has led to this study's aim: to extract the essential oil from MS and analyze its potential to improve sleep. This study utilized petroleum ether for the thermal extraction of the essential oil from MS, which was then subjected to compositional analysis using Gas Chromatograph Mass Spectrometer (GC–MS). P‐chlorophenyl alanine (PCPA) was used to induce an insomnia model in Sprague–Dawley (SD) rats. Following the successful establishment of the model, the MS essential oil was administered at concentrations of 10%, 5%, and 2.5% to investigate its sedative and hypnotic effects. The efficacy of the MS essential oil was assessed by observing the general condition of rats in each group, conducting an open field test, a pentobarbital sodium righting test, and measuring the serum 5‐HT (5‐hydroxytryptamine) levels and hypothalamic GABA (γ‐aminobutyric acid) content. GC–MS analysis of the MS essential oil revealed a rich composition, including oleic acid, palmitoleic acid, stigmasterol, and γ‐stigmasterol, among other substances. Through the assessment of the rats' general condition, behavioral tests, and blood biochemical assays, it was inferred that MS essential oil aromatherapy can reduce the rat's locomotor activity, increase their interest in activity and exploration, enhance the serum 5‐HT levels, and elevate hypothalamic GABA content. Consequently, it can be concluded that MS essential oil has a sedative and hypnotic effect.

## Graphical abstract

The main components of *Moringa seed* essential oil include 15 compounds such as oleic acid, palmitoleic acid, and stigmasterol. *Moringa seed* essential oil improves sleep by increasing GABA in the hypothalamus and reducing 5‐HT levels in serum through the olfactory pathway in rats. After aromatherapy intervention, the sleep latency of the rats in the *Moringa seed* essential oil group was decreased; the sleep time was increased; the anxiety and tension were improved; and the autonomic activity and exploration ability of the rats were increased.

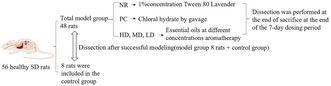

## 1 INTRODUCTION

Insomnia is a common and widespread clinical disorder, characterized by difficulty in falling asleep, frequent awakenings during the night, and challenges in returning to sleep after waking up (Brownlow et al., 2020). Chronic insomnia can progressively lead to physiological and pathological changes in patients, potentially resulting in cognitive impairment. In severe cases, it is often accompanied by anxiety or depression, which can have a significant adverse impact on their daily lives (Jiahui et al., 2021; Mengjiao, 2022; Yingzi et al., 2022). As society has evolved, the issue of insomnia has become increasingly widespread. Studies have indicated that the prevalence of insomnia disorder ranges from approximately 19% to 50% (Guadagna et al., 2020; Sutton, 2021). Sedative, hypnotic, and antidepressant medications are commonly used to enhance patients' sleep quality. However, these medications can be costly and may not be easily accessible. Moreover, prolonged use can result in drug dependence, as well as side effects such as somnolence, dizziness, and fatigue (Jodaki et al., 2021; Read & Williams, 2018). Thus, it is evident that the use of the aforementioned pharmacological treatments not only imposes an economic burden on patients but also restricts their potential for widespread application to a certain degree (Tang et al., 2021). Hence, it is imperative to identify a treatment for insomnia that is cost‐effective, efficient, and carries a low risk of adverse effects.

Aromatherapy represents a distinctive treatment approach that is widely used because it is safer compared to oral medications and avoids first‐time elimination and gastrointestinal irritation (Agatonovic‐Kustrin et al., 2020; Karadag et al., 2017). Research has shown that essential oils from aromatic plants can have beneficial sedative and hypnotic effects. For instance, *Lavandula angustifolia*, which is rich in volatile oils derived from the aromatic components of the plant, is regarded as one of the most potent over‐the‐counter aromatic herbal extracts for alleviating anxiety, depression, and stress (Lopez et al., 2017; Sanchez‐Vidana et al., 2019; Wotman et al., 2017). Furthermore, the volatile oil extracted from *jujube kernel* using supercritical CO_2_ technology has been found to exhibit sedative and hypnotic properties (Hwang & Shin, 2015). Inhalation of *Roman chamomile*, when combined with clomipramine treatment, has been shown to enhance the efficacy of treatment for depression‐like behavior that is resistant to conventional therapy in mice (Ebrahimi et al., 2022; Hashikawa‐Hobara et al., 2019; Jia et al., 2021). *Rose* essential oil has anxiolytic and antidepressant effects (Kunduo et al., 2018; Matias Nascimento Maia et al., 2021). In a study conducted by Tingting Lu, it was demonstrated that *Pelan* essential oil possesses sedative and hypnotic properties (Tingting et al., 2018). Dolzhenko discovered that *Lavandula* essential oil (Silexan) capsules have sedative and hypnotic effects. Additionally, Wenzhuo's research confirmed that *bergamot lactone* has positive effects on neurotransmitters and enhances learning and memory in rats with PCPA‐induced insomnia (Perna et al., 2019; Wenzhuo et al., 2020; Yap et al., 2019). It follows that plant essential oils had some effectiveness in the treatment of insomnia.

*Moringa oleifera* is a perennial, tropical deciduous tree belonging to the genus *Moringa* within the family Moringaceae. It is extensively cultivated in tropical and subtropical regions across Asia and Africa (Pareek et al., 2023). Moringa seeds refer to the seeds of the Moringa tree, which are not only directly edible but also hold significant nutritional value (Dhakad et al., 2019). Research has indicated that Moringa seeds (MS) are abundant in oils (comprising a high concentration of unsaturated fatty acids and up to 70% more oleic acid), proteins (high in protein content, four times that found in milk), polysaccharides, *Moringa xanthones*, polyphenolic compounds, and various vitamins, among other nutrients. Additionally, MS are known to have medicinal properties. Current research on MS primarily focuses on the effects of the seeds in their entirety, such as its neuroprotective effects against diabetic encephalopathy in rats (Jiang et al., 2022), the effect of aqueous extract on alcohol‐induced motor dysfunction in nematodes (Ying, 2020), and so on. According to the Indian Pharmacopeia of Ayurveda, MS are recommended for the treatment of neurological disorders (Parasuraman et al., 2014), and insomnia is believed to stem from an imbalance in the regulation of activity between the sympathetic and parasympathetic nervous system (Stein & Pu, 2012). Meanwhile MS are known for their high oil and volatile oil content. Zou Yu, in a comparative study of two extraction methods for MS oil, found that the volatile oil yield from petroleum ether thermal extraction was 25.5%. Additionally, the samples extracted using petroleum ether thermal extraction exhibited a more pronounced oil aroma and a distinct roasted coffee scent (Yu et al., 2011). Volatile oils, which are active compounds derived from aromatic herbs, frequently possess a broad spectrum of biological activities (Wang et al., 2014). In conclusion, the question of whether the volatile oil of MS can induce a sedative and hypnotic effect, particularly when combined with aromatherapy, remains an area of theoretical consideration. Simultaneously, the impact of MS on sleep is still in the preliminary stages of exploration. Shuxiang Qin has reported on the hypnotic effects of MS ingredients, suggesting that there is a basis for further investigation into their potential therapeutic applications (Qinshuxiang et al., 2016). Recent studies have demonstrated that chemically active constituents found in essential or volatile oils can exert neuroprotective effects and may assist in mitigating symptoms of depression and anxiety (Cui et al., 2022). However, MS have been less studied in insomnia.

Therefore, the objective of this study was to extract the essential oil from MS and investigate its potential to enhance sleep quality, as well as to identify the factors contributing to its efficacy. This research aims to provide a foundation for the further development of MS and, based on the findings, to develop a hypnotic essential oil from MS.

## 2 MATERIALS AND METHODS

### 2.1 Main materials and instruments

MS (Database ID: MY‐4096) were obtained from Yuanjiang County, Yunnan Province. Petroleum ether, chloral hydrate (Tianjin Kemiou Chemical Reagent Co., Ltd., Beijing, China), P‐chloro phenylalanine (Shanghai Macklin Biochemical Technology Co., Ltd., Beijing, China), polysorbate‐80 (Wuhan Tianzhi Biotechnology Co., Ltd., Beijing, China), Mm EDTA, 0.9% saline (Guizhou Colun Pharmaceutical Co., Ltd., Beijing, China), 75% alcohol (Guizhou Xinyuan Biotechnology Co., Ltd., Beijing, China), GABA ELISA kit (Nanjing Jiancheng Institute of Biology, Beijing, China), lavender machine (Shenzhen You Industrial Group Co., Ltd., Beijing, China), vacuum drying oven (Shanghai Qixin Scientific Instrument Co., Ltd., Beijing, China), multipurpose thermostatic ultrasonic extraction machine (Jiangsu Tianling Instrument Co., Ltd., Beijing, China), rotary evaporator (Shanghai Yarong Biochemical Instrument Factory, Beijing, China), constant temperature shaker (Shanghai Tiancheng Experimental Instrument Manufacturing Co., Ltd., Beijing, China), high‐speed refrigerated centrifuge (Shanghai Anting Scientific Instrument Factory, Beijing, China).

### 2.2 Experimental methods

#### 2.2.1 Extraction of essential oil from MS, component analysis

100 g MS were removed in a drying oven with removed heteroplasmy and dehulled at 80°C, after drying for 4 h, cooled to room temperature, and put into a grinder and pulverized through a 40 mesh sieve. Add petroleum ether in a material to a liquid ratio of 1:10, sonicate for 40 min in an ultrasonic cleaning device, stir and extract at 40°C and 168 *g* in a constant temperature heating magnetic stirrer for 45 min, then filter and extract the filtrate. The filtrate was put into the rotary evaporator for rotary concentration to obtain *Moringa oleifera* oil (Yang et al., 2019).

GC–MS analysis of *MS* oil: Component analysis was conducted using GC–MS and NIST11. LIB perform component analysis using a standard library. The conditions were as follows (Yang et al., 2019):

GC conditions: 19091 S‐433 HP‐5 MS (5% Phenol Metal Siloxme, 30 m × 250 μm × 0.25 μm) chromatographic column. The heating program is to maintain a column temperature of 50°C for 1 min, 5°C/min for heating, 180°C for 2 min, 5°C/min for heating, and 290°C for 10 min. The vaporization chamber temperature is 250°C, the injection volume is 1.0 μL, the split ratio is 10:1, and the carrier gas (He, 99.999%) flow rate is 1 mL/min.

MS conditions: EI ion source, ionization voltage 70 eV, ion source temperature 230°C, fourth pole temperature 150°C, interface temperature 250°C.

### 2.3 Establishing a rat insomnia model

#### 2.3.1 Experimental animals and groups

Fifty‐six healthy SD rats, with a body weight of (230 ± 50) g, half male and half female, were fed adaptively for 7 days at a room temperature of 20 (±1)°C, fed with regular feed, drank freely, kept the bedding dry and the environment quiet. After numbering the rats, they were randomly divided into seven groups according to a random number table, including a control group, a PCPA model group, a no‐dose natural recovery group (NR), a chloral hydrate positive control group (PC), a high‐dose group (HD), a medium‐dose group (MD), and a low‐dose group (LD) (with concentrations of 10%, 5%, and 2.5%, respectively, and a large concentration gradient was set according to the reference literature and initial exploration), with eight rats in each group, half male and half female. Among them, the control group was compared with the model group to explore whether the modeling was successful; the NR was compared with the treatment group to explore whether the treatment was effective.

#### 2.3.2 Insomnia rat model preparation

Before modeling, the rats were weighed, and PCPA was prepared as a suspension with weakly basic saline according to the literature at 450 mg/kg, and the rats in the model group were injected intraperitoneally (10 mL/kg) at 12:00–13:30 daily for 2 days, except for control group. Weigh the weight of rats at a fixed time every day and observe their changes.

At the end of modeling, eight rats in the control group and eight rats in the model group were dissected through their general conditions, data from the open field test, and data from the sodium pentobarbital righting reflex test, and if they were successful, their tissues were placed in a –80°C freezer for examination; if unsuccessful, continue to inject PCPA suspension until success starts subsequent trials.

### 2.4 Animal administration

**FIGURE 1:**
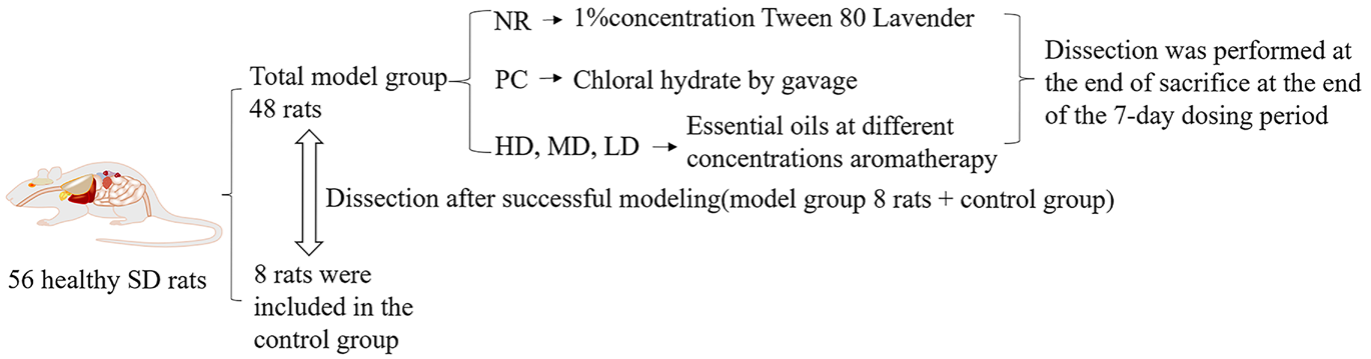
Grouping, modeling, and administration flowchart.

MS essential oil at 10%, 5%, and 2.5% concentrations were placed into the ultrasonic atomized lavender to open the ultrasonic atomized lavender so that it diffused throughout the chamber, and rats in the three dosing groups were placed into the smoky chamber sequentially and covered with a box cover every day from 1:00 to 2:00 pm for 1 h continuous inhalation. The control group was not smoked with the positive control group, NR was given 1% solution of Tween 80 in ultrasonic aerosolized smoked lamps, and after the rats had acclimated for 5 min, the smoked lamps were turned on, covered with a smoked chamber cover, and sniffed for 1 h, and smoked for 7 days (Yingzi et al., 2022). PC was treated with chloral hydrate at a dose of 200 mg/kg according to the LD50 of mice (Beibei et al., 2022) and then calculated by the equivalent dose converted according to body surface area among animals for 7 days (Beibei et al., 2022) (As shown in Figure 1).

### 2.5 Experiments and observation of indicators

#### 2.5.1 General condition of each group after drug administration

Rats in each group were observed for hairy gloss or not after smoking for 7 days, mental status, and body weight after 7 days of treatment (Libo et al., 2020; Wenzhuo et al., 2020; Xiaoye et al., 2020).

#### 2.5.2 Open field test

Rats in each group were subjected to an open field test 30 min after the final aromatherapy or drug administration. Rats were acclimatized for 5 min, and behavioral parameters were counted over 4 min and compared between rats in each group. At the end of each rat's test, the rat's feces were cleaned, and the rat odor was removed by wiping it after spraying with 75% alcohol so as not to affect subsequent tests.

Recording indices: voluntary activity test index: total locomotion distance (cm); maximum velocity (cm/s); average velocity (cm/s); rest time (seconds); number of arm lifts (Zhong et al., 2019).

#### 2.5.3 Sodium pentobarbital righting reflex test

Pentobarbital sodium was administered intraperitoneally at the end of modeling and 36 h after the last dose, based on a dose of 40 mg/kg and an injection volume of 10 mL/kg. The rat righting reflex disappeared, that is, the rat maintained the position of the back down for more than 30 s, and the eyelid reflex disappeared, the time was the latency to fall asleep, and the time that the righting reflex disappeared to wakefulness, that is, was conveyed three times with 30 s varus as an indicator of the end of sleep, was the duration of sleep. Sleep latency as well as the time at which the righting reflex disappeared were recorded.

Recording indices: the rats in each group were given intraperitoneal injections, and the time to fall asleep and wake time, and their sleep latency and sleep duration were calculated, respectively (Libo et al., 2020; Wenzhuo et al., 2020; Xiaoye et al., 2020).

### 2.6 Sample collection

Two days after dosing, anesthetize all rats by injecting pentobarbital sodium. Blood was collected from the abdominal aorta, and centrifuged at 1509 *g* for 20 min, the supernatant was removed and stored in the freezer at –80°C for further use; then the rats were sacrificed, their heads rapidly dissected on ice, the whole brains were removed, and a part of the hypothalamic tissue was isolated and placed into a 2 mL EP tube pre‐coated with tin paper and filled with 4% paraformaldehyde fixative for HE staining. The rest was quickly separated from the hypothalamus and washed in ice normal saline, put into a 2 mL EP tube, do the mark, and stored in the freezer at –80°C for detection of 5‐HT in serum and GABA in the hypothalamus. The study was approved by the Ethics Committee for the animal care welfare committee of Guizhou Medical University (NO: 2200385).

### 2.7 H&E staining to detect hypothalamic histopathological changes

#### 2.7.1 Tissue paraffin‐embedded sections

Extraction: freshly removed fresh tissues were fixed in 4% paraformaldehyde for more than 24 h. Transfer fresh tissue from the fixation.

The fixative was removed, and the destination site tissue was cut flat with a scalpel in a fume hood, with the trimmed tissue and advance‐ready tags placed inside the dehydration box in preparation for dehydration.

Dehydration: the dehydration box was put into the hammock in the dehydrator in a sequential gradient of alcohol for dehydration. Put hypothalamus in 75% alcohol for 4 h and take it out. Add 85% alcohol for 2 h and take it out. Add 90% alcohol for 2 h and take it out. Add 95% alcohol for 1 h and take it out. Add anhydrous ethanol I for 30 min and anhydrous ethanol II for 30 min and take it out.

Embedding: wax‐soaked tissues are processed for embedding within an embedding machine. Thawed wax was first placed into the embedding box, and the tissue was removed from the dehydration box before the paraffin was solidified, placed into the embedding box as required for the embedding surface, and labeled accordingly. Place the embedding frame to the embedding machine's right side ice to cool, pending wax solidification, gently tap the wax block out of the embedding frame, and trim the wax block edges.

Sections: trimmed wax blocks were sectioned on a paraffin microtome to a slice thickness of 4 μm. Sections were floated on a slide maker with 40°C warm water to spread the tissue flat, stack the tissue up with glass slides, and put into a 60°C oven for baking.

#### 2.7.2 Staining of sections

Dewaxing and rehydration of paraffin sections: first put it into xylene I 20 min, then xylene II 20 min, then absolute ethanol I 5 min, then absolute ethanol II 5 min, then 75% ethanol 5 min, and finally wash with tap water.

Hematoxylin staining: sections were put into hematoxylin staining solution for 3–5 min, washed in tap water, differentiated in differentiation solution, washed in tap water, refluxed blue solution, and rinsed in running water.

For eosin staining, sections were dehydrated in a graded alcohol series of 85%, and 95% for 5 min each, and embedded in an eosin staining solution for 5 min.

Dehydration and sealing: put the slices into absolute ethanol I for 5 min, then take them out in absolute ethanol II for 5 min, then put them into absolute ethanol III for 5 min, then put them into xylene I for 5 min, then put them into xylene II for 5 min, and finally seal them with neutral balsam.

Place the stained tissue sections under the microscope for observation, capture images, and conduct analysis.

### 2.8 Determination of 5‐HT and GABA content

Accurately weigh the animal tissue weight in weight (mg): Volume (μL) = 1:9 add 9 volumes of 0.9% saline, mechanically homogenize in ice water bath conditions, prepare into 10% homogenate, 3000 rpm, centrifuge for 10 min, and remove supernatant for determination.

Loading: add 100 μL of the appropriate dilution of the sample to be examined to the previously coated reaction wells described above.

Incubation: incubate at 37°C for 1–2 h after plate sealing with sealing membrane.

Washing: carefully unmask the sealing plate membrane, place it in a plate washer, and wash 3–5 times. Plates can also be washed manually: discard liquid, add 300 μL wash solution per well, soak for 1–2 min, pat dry on absorbent paper, and repeat 3–5 times.

Plus‐antibody: 100 μL of diluted biotinylated antibody working solution was added to each well.

Incubation: incubate at 37°C for 1 h after plate sealing with sealing membrane.

Washing: carefully unmask the sealing plate membrane, place it in a plate washer, and wash 3–5 times. Plates can also be washed manually: discard liquid, add 300 μL wash solution per well, soak for 1–2 min, pat dry on absorbent paper, and repeat 3–5 times.

Plus‐enzyme conjugate: 100 μL of diluted enzyme conjugate working solution was added to each well.

Incubation: after the plate was sealed with a sealing membrane, it was incubated at 37°C in the dark for 30 min.

Washing: carefully unmask the sealing plate membrane, place it in a plate washer, and wash 3–5 times. Plates can also be washed manually: discard liquid, add 300 μL wash solution per well, soak for 1–2 min, pat dry on absorbent paper, and repeat 3–5 times.

Color development substrate: 100 μL of TMB substrate solution was added to each well and allowed to react for 10–30 min at 37°C in the dark until a distinct color gradient appeared in the wells with the standards diluted several times.

The reaction was stopped: 100 μL of 2 M sulfuric acid was added to each reaction well, and the color changed from blue to yellow.

Results measurements: OD values of each well were measured within 10 min at 450 nm on a microplate reader after zeroing the control group wells.

### 2.9 Data analysis

Graphpad8.0 software was applied for statistical analysis and mapping. All indexes were measured as mean ± standard deviation ($\bar{x}$ ± s) was used as described, values of each index were tested for normality and homogeneity of variance, and if normality and homogeneity of variance were followed by one‐way ANOVA followed by LDS for comparison between groups; non‐parametric tests were used if groups did not meet normality. Where *p* < .05, *p* < .01 indicated that the difference was statistically significant.

## 3 RESULTS

### 3.1 MS essential oil extraction yield, component analysis

**FIGURE 2:**
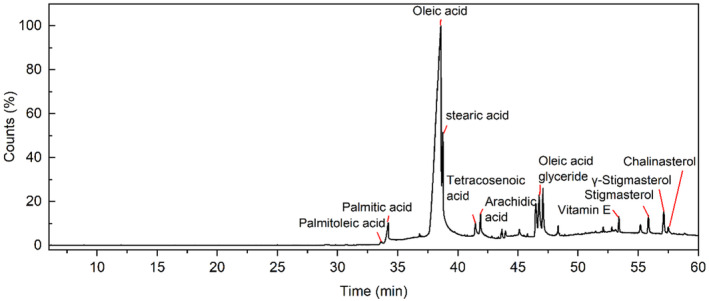
GC–MS mass spectrometry.

An essential oil yield of 22.08 ± 0.7 g was obtained from 100 g of Moringa seeds (MS), corresponding to an extraction yield of 22.08%. The primary constituents of the MS essential oil, as determined by Gas Chromatography–Mass Spectrometry (GC–MS), included 76.46% oleic acid, 6.96% stearic acid, 3.44% palmitic acid, 3.28% oleic acid glyceride, and 2.05% γ‐stigmasterol. These components are detailed in Figure 2 (Component can be found in Table 1).

**TABLE 1:** Main components of MS essential oil.

Chemical compound	Chemical formula	Peak area	Molecular weight	Ions	(%)
Palmitoleic acid	C_16_H_30_O_2_	431865457	254.41	236, 97.83	0.61
Palmitic acid	C_16_H_32_O_2_	2344658323	256.42	256, 129, 73, 213	3.44
Oleic acid	C_18_H_34_O_2_	52052013547	282.46	264, 55, 212	76.46
Stearic acid	C_18_H_36_O_2_	4737220436	284.48	284, 129.73	6.96
Tetracosenoic acid	C_20_H_38_O_2_	1119931252	310.51	368, 129.73	1.65
Arachidic acid	C_20_H_40_O_2_	1460553108	312.53	312, 129.73	2.15
Linoleic acid ester	C_21_H_38_O_3_	214370197	338.52	55, 129, 185, 265	0.31
Oleic acid glyceride	C_21_H_40_O_4_	2233880768	356.54	264, 55.98	3.28
Vitamin e	C_29_H_50_O_2_	772100045	430.50	430, 165, 205	1.13
Stigmasterol	C_29_H_48_O	935164585	412.69	255, 394.83	1.37
γ‐Stigmasterol	C_29_H_50_O	1393807700	414.71	414, 213, 107	2.05
Chalinasterol	C_28_H_46_O	396178175	398.66	314, 271, 299	0.58

### 3.2 Intervention of MS essential oil on insomnia rats

#### 3.2.1 Modeling results

**FIGURE 3:**
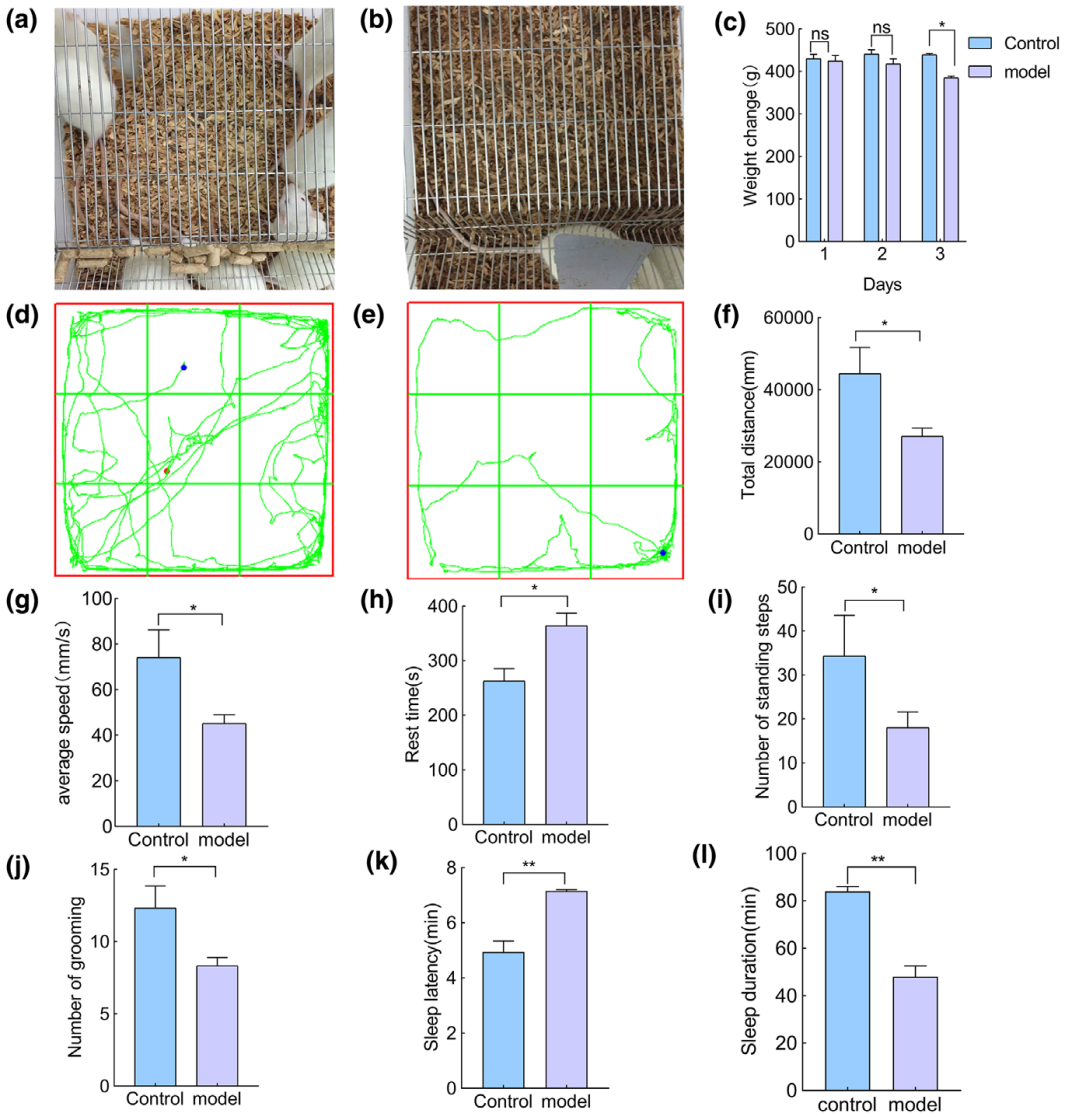
The modeling results: (a) the pad for rats in the control group, and (b) the pad for the model group. (c) The rat body weight change graph, (d) the control group action trace, (e) NR action trace, (f) the total water distance, (g) the average velocity, (h) the resting time, (i) the number of standing times, (j) the number of grooming times, (k) rat sleep latency and (l) rat sleep time. Where * indicates a significant difference compared with the control (*p* < .05), ** indicates a significant difference compared with the control group (*p* < .01).

As shown in Figure 3a,b, following the successful establishment of the model, the bedding in the model group appeared more moist compared to the blank control group.

As shown in Figure 3c, weight trend analysis indicated that the control group exhibited normal fluctuations in body weight after modeling, whereas the model group showed a downward trend in weight. The body weight of the model group significantly decreased compared to the control group (*p* < .05). Additionally, the success of the model was assessed through behavioral changes, as measured by the open field test and the pentobarbital sodium righting reflex test. Upon completion of the open field test, the rat trajectories shown in Figure 3d,e revealed that the rats in the control group were more active than those in the model group. The movement data for both groups of rats, as presented in Figure 3f–j were analyzed and revealed that the total distance moved (*p* < .05), average speed (*p* < .05), frequency of rearing (*p* < .05), and frequency of grooming (*p* < .05) were significantly lower in the model group rats compared to the control group. Conversely, the stationary time (*p* < .05) of the model group rats was notably higher than that of the control group.

As shown in Figure 3k,l, the sleep latency of the rats in the model group was significantly longer compared to the control group (*p* < .05), and the total sleep time of the model group rats was significantly shorter than that of the control group (*p* < .05). These findings suggest that the sleep cycle of the rats in the model group was disrupted by the pharmacological intervention, further confirming the successful establishment of the insomnia model.

#### 3.2.2 Intervention outcomes

##### Changes in general indicators

**FIGURE 4:**
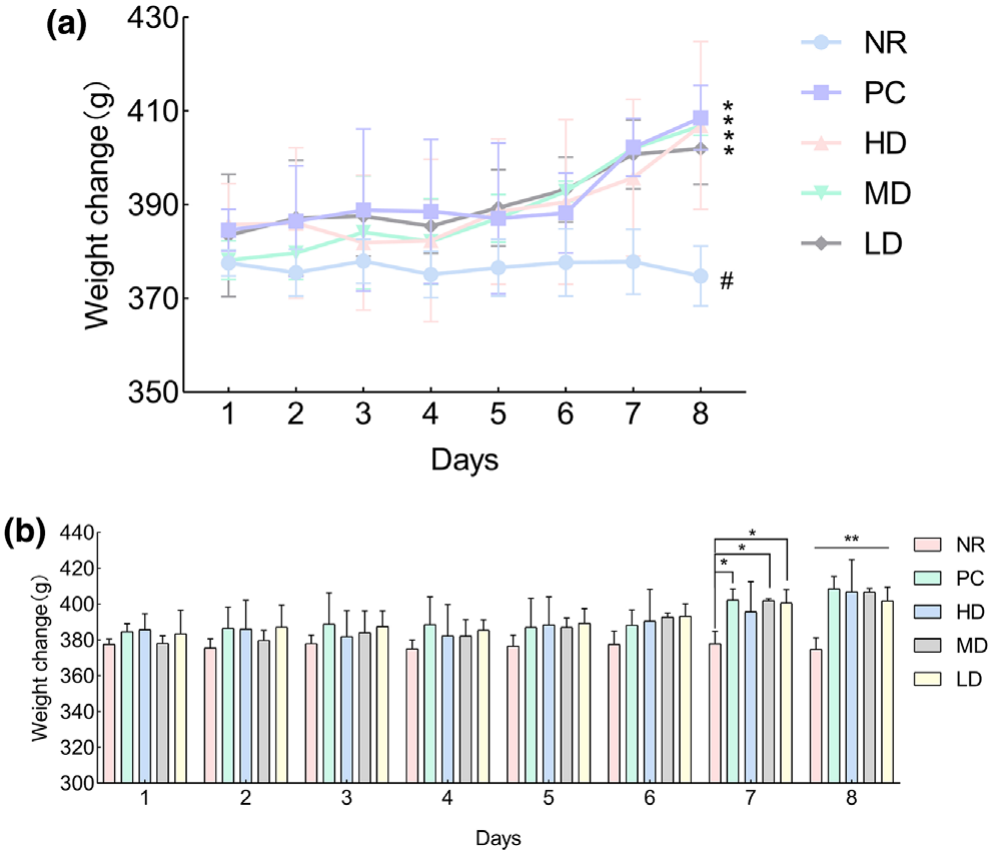
General physical changes: (a) It is a trend chart of weight change. In (a), * indicates an upward trend, and # indicates a downward trend. (b) It is a weight change chart. In (b), * represents a significant difference compared to the natural recovery group. Where * indicates a significant difference compared with NR (*p* < .05), ** indicates a significant difference compared with NR (*p* < .01).

During the administration period, the weight of rats was measured and recorded at a fixed time every day, and the drug effect was preliminarily determined based on the weight. As shown in Figure 4a, the weight of the rats in the treatment group exhibited a significant upward trend following administration, whereas the weight of the NR group showed a downward trend (further analysis can be found in Figure S1 of the Appendix S1). As shown in Figure 4b, by the 6th day of administration, the NR group exhibited a significant weight loss (*p* < .05); by the 7th day of administration, the PC, HD, MD, and LD groups showed statistically significant differences in body weight compared to the NR group (*p* < .05, *p* < .05, *p* < .05).

##### Comparison of open field test indicators after administration

**FIGURE 5:**
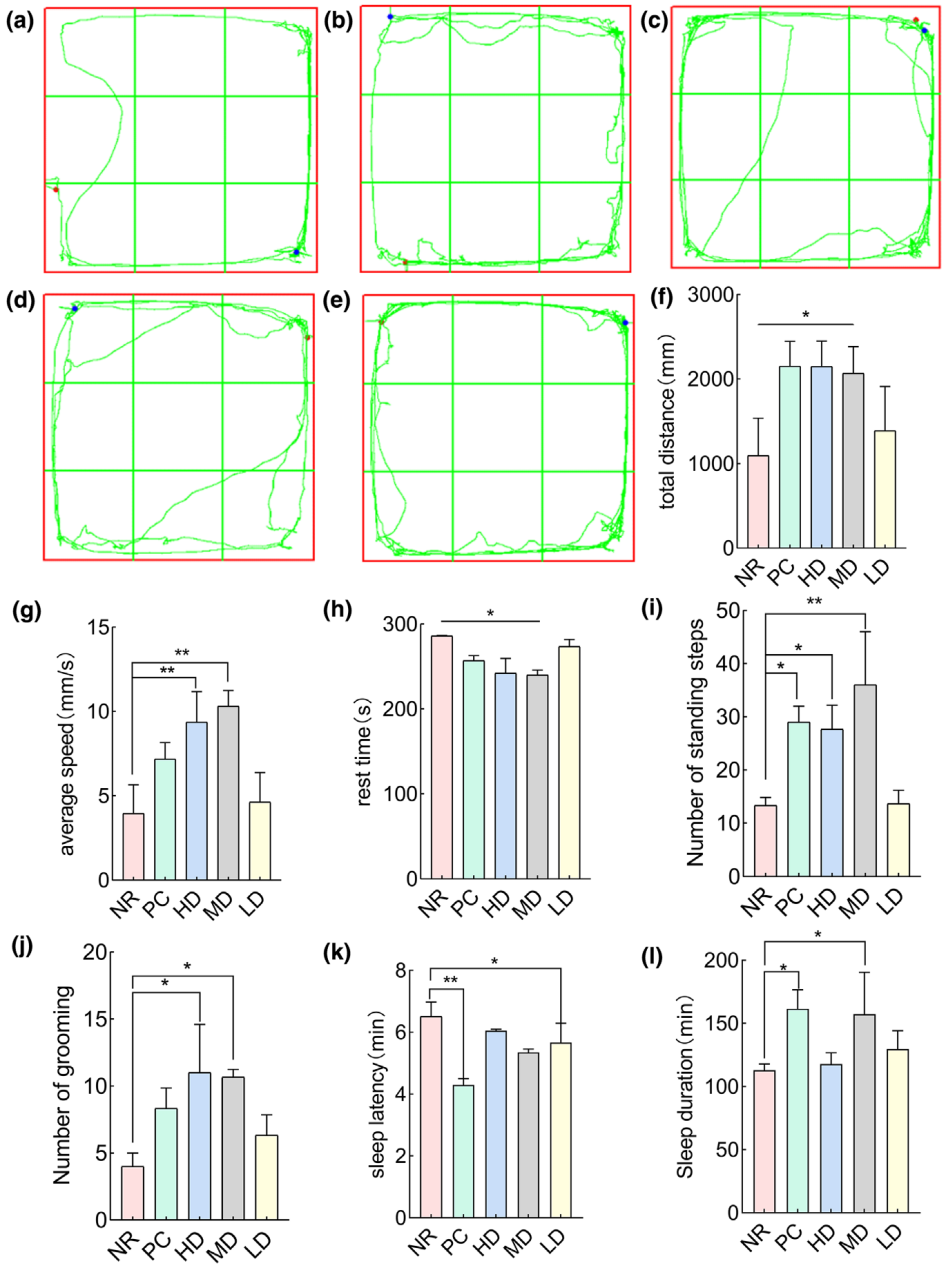
Behavioral experiments: (a) NR, (b) PC, (c) HD, (d) MD, and (e) LD. (f) The total water distance, (g) the average velocity, (h) the resting time, (i) the number of standing times, and (j) the number of grooming times. (k) Rat sleep latency and (l) rat sleep time. Where * indicates a significant difference compared to the natural recovery group (*p* < .05), and ** indicates a significant difference compared to the natural recovery group (*p* < .01).

As shown in Figure 5a–e, the trajectories from the open field test after administration indicate that compared to the NR group, the activity levels of PC, HD, MD, and LD groups have increased, with a higher frequency of exploration in the central grid area.

As shown in Figure 5f, the total exercise distance of the PC, HD, and MD groups was significantly greater than that of the NR (natural recovery) group (*p* < .05). Although there was no significant difference in the total exercise distance of the LD group compared to the NR group, the overall trend was higher than that of NR. As shown in Figure 5g, the average exercise speed of the PC, HD, and MD groups was significantly higher than that of the NR group (*p* < .05). Although there was no significant difference in the average exercise speed of the LD group, it did demonstrate an increase in the average speed of the rats. As shown in Figure 5h, the rest time for the PC, HD, and MD groups was significantly longer compared to that of the natural recovery group (*p* < .05). Although there was no significant difference in resting time between the LD group and the NR group, the resting time of the rats in the LD group increased. As shown in Figure 5i, the number of standing times during exercise in the PC, HD, and MD groups was significantly higher than that during the NR group (*p* < .05). Although there was no significant difference in the LD group, it increased the number of standing times in rats. As shown in Figure 5j, the frequency of grooming during exercise in the HD and MD groups was significantly higher than that in the NR group (*p* < .05). Although there was no significant difference in the frequency of grooming during exercise between PC and LD, there was an observed increase in the grooming frequency among the rats, with the PC group showing a stronger trend of increase compared to the LD group.

As shown in Figure 5k, compared with NR, the sleep latency in the PC group (*p* < .05) and the MD group (*p* < .05) was significantly reduced. Although there was no significant difference between the HD and LD groups, the sleep latency of rats in both concentration groups showed a decrease compared to the NR group.

As shown in Figure 5l, compared with the NR group, the sleep time of the PC and MD groups was significantly increased (*p* < .05). Although there was no significant difference between the HD and LD groups, the sleep time of rats in both the HD and LD groups increased, with the LD group showing a stronger trend of increase than the HD group.

##### Histopathological changes and neurotransmitter detection after administration

**FIGURE 6:**
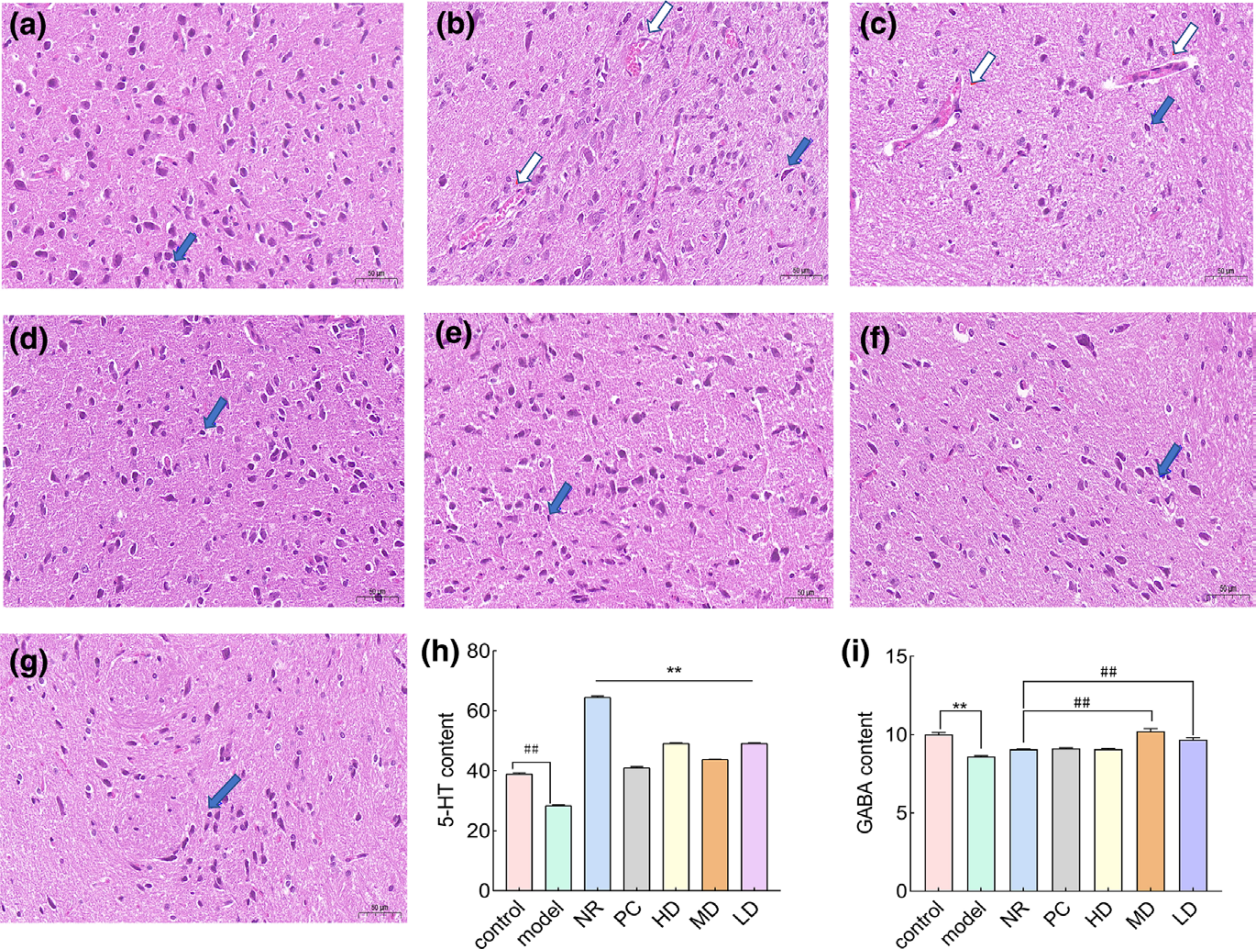
HE staining (40×), tissue testing, and blood biochemical test results: (a) the control group, (b) the model group, (c) NR, (d) PC, (e) HD, (f) MD, and (g) LD. Blue arrows were hyperchromatic for nerve cell pyknotic nuclei and white arrows were small focal hemorrhages. (h) The 5‐HT content of the control group versus the model group expressed by ##, where ## indicated a significant difference (*p* < .01*) compared with the control group. The 5‐HT content of the positive control group versus the three concentration groups with natural recovery is indicated by **, where ** indicates a significant difference (*p* < .01*) compared with the natural recovery group. (i) The GABA content of the control group versus the model group indicated by **, where ** indicates a significant difference (*p* < .01#). The GABA content of the positive control group versus the three concentration groups with natural recovery was expressed by ##, where ## indicates a significant difference (*p* < .01).

As shown in Figure 6a–g, the H&E staining results were magnified 40 times. It can be observed that, compared to the model group, the control group exhibited a small number of neural cell nuclei with proper fixation and deep staining, devoid of any other abnormalities. In contrast, the model group and NR occasionally displayed small focal hemorrhages, shallow cell staining, and numerous nerve cell nuclei with deep staining. The PC, HD, MD, and LD groups showed normal staining of nerve cells. When compared to NR, their nerve cell nuclear fixation and deep staining were relatively reduced, and the bleeding points were also smaller than those of NR. Overall, the model group and NR showed more severe abnormalities, followed by the PC, HD, MD, and LD groups, while the control group exhibited no abnormalities.

As shown in Figure 6h, the serum levels of 5‐HT revealed that the control group had a significantly higher 5‐HT content than the model group (*p* < .01), suggesting the successful establishment of the model. In contrast, the NR group displayed significant abnormalities in comparison to the other concentration groups.

As shown in Figure 6i, the content of GABA in the hypothalamus showed that the GABA content in the control group was significantly higher than that in the model group (*p* < .01), indicating the successful establishment of the model. Compared with NR, the GABA content in MD and LD was significantly higher than that in NR (*p* < .01, *p* < .01).

##### Correlation analysis

**FIGURE 7:**
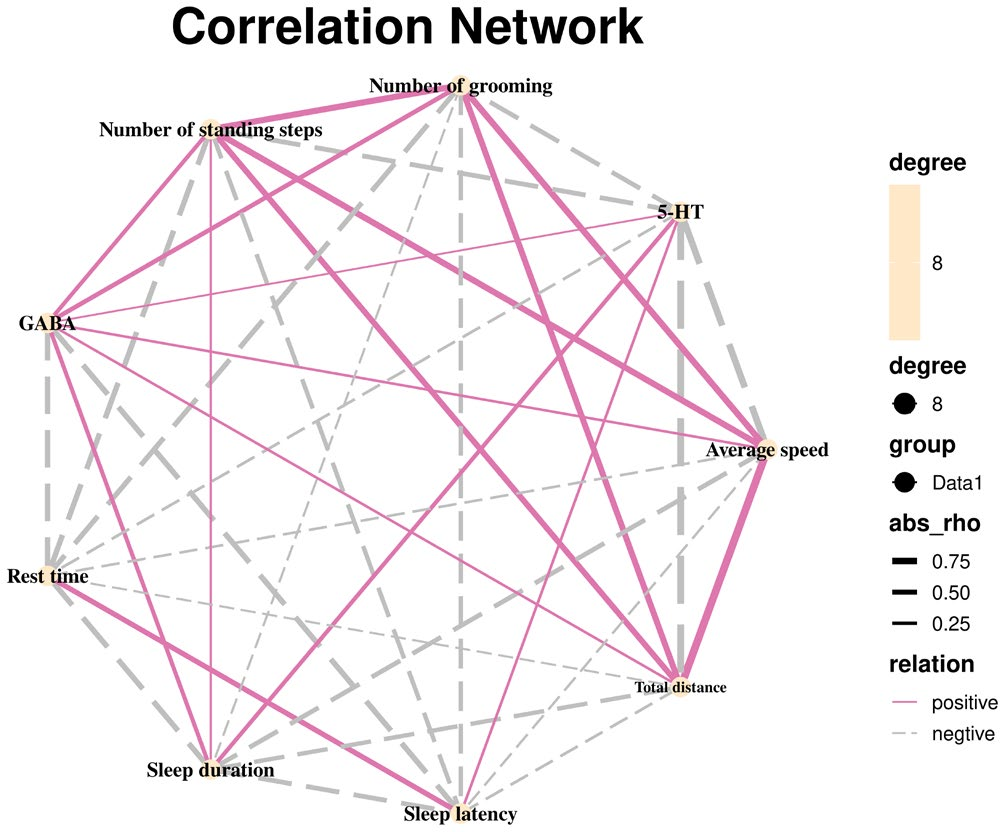
Correlation analysis.

As shown in Figure 7, the total distance traveled, mean velocity, number of standing bouts, and grooming bouts in the open field test were all positively correlated with GABA levels in the hypothalamus. There was a negative correlation between the time spent at rest these variables, as well as with GABA in the hypothalamus, 5‐HT in the serum, and sleep duration in the pentobarbital sodium righting reflex test. GABA levels in the hypothalamus were positively correlated with 5‐HT in the serum and negatively with sleep latency in the sodium pentobarbital righting reflex experiment. The time spent sleeping in the pentobarbital sodium righting reflex test was positively correlated with the number of rearing bouts in the open field test, while the 5‐HT in serum was negatively correlated with the total distance traveled, average speed, and several grooming bouts. Sleep latency was positively correlated with resting time in the open field test and 5‐HT in serum.

## 4 DISCUSSION

Insomnia, a common disease in the current population, is being treated with essential oil aromatherapy. This method offers benefits such as safety, reliability, low cost, ease of use, and high safety (Agatonovic‐Kustrin et al., 2020). It can be used as an auxiliary or alternative medication to treat insomnia. The seeds of *Moringa* tree can be used to treat nervous system disease according to the Indian Ayurveda Pharmacopeia (Parasuraman et al., 2014), while insomnia is considered a condition caused by an imbalance in the regulation of activities between the sympathetic and the parasympathetic nervous system. Therefore, this study extracted MS essential oil, investigated its potential active compounds, and examined its efficacy in improving sleep.

Oleic acid and sterols have a certain effect on improving sleep, such as studies by Irmisch et al. (2007). Palmitic acid and oleic acid are especially significant in the treatment of sleep disorders. The possible mechanism of action is that they act as precursors to oleamide, which has a sleep‐inducing effect. Medina‐Rodriguez et al. (2020) discovered that oleic acid acts as an AI‐2 inhibitor and possesses antidepressant properties, which can help depressive behavior. In addition, GHOSH S (Ghosh et al., 2022) confirmed the antidepressant‐like effects of the total sterol component and stigmasterol in the leaves of Angelica. This study used the petroleum ether thermal extraction method to extract essential oil from *Moringa oleifera* seeds and analyzed its components with GC–MS. The primary constituents of MS essential oil are oleic acid, palmitic acid, stigmasterol, and other substances. Next, this study preliminarily investigates whether the inhalation of MS essential oil through the olfactory pathway can effectively contribute to improving sleep disorders.

Bian et al. (2021) research shows that intraperitoneal injection of PCPA can induce insomnia in rats. Approximately 48 h after injection of PCPA, most rats start to display symptoms similar to ADHD. In addition to behaviors similar to ADHD, the mice also exhibited increased sensitivity and aggression, dry and dull fur, consumed more water, reduced food intake, and had loose stools, leading to weight loss (Mengjiao, 2022). Therefore, in the initial phase of this study, an insomnia model in rats was established, by intraperitoneal injection of PCPA suspension. The success of modeling was determined by observing changes in hair color, the moisture level of the rat bedding, and fluctuations in body weight. Under the influence of the drug, the sleep cycle of the rats was disrupted, resulting in yellowing and loss of luster in their fur. At the same time, a reduction in diet and an increase in water intake led to increased urination and loose stools. Therefore, the model group bedding is wetter than the control group. The decrease in diet and increase in drinking water resulted in a significant weight loss in the model group, while the weight of the control group remained unchanged or slightly increased, consistent with the literature description. It is preliminarily judged that the modeling was successful. After administering the treatment medication in the later stage, their general condition was observed. Rats in the NR group displayed irritability, defensiveness, resistance, and mutual biting, while the treatment group did not exhibit any obvious biting behavior. Observing the weight changes of rats, the positive control group and the three treatment groups with different concentrations show an upward trend in body weight. Meanwhile, the NR group still exhibits a downward trend in body weight, with the HD and MD groups displaying the most significant weight changes. Based on the general changes in the condition of rats, it is tentatively concluded that the medication was effective. Insomnia induces depression‐like behavior in animals (Chang et al., 2014), therefore, we can assess depressive‐like behaviors to judge the sleep condition of the animals through behavioral assessments.

The open field test is a classic emotion‐related behavioral test used to detect the behavior of animals in unfamiliar environments, including spontaneous activity and exploratory behavior. It is a widely employed technique for assessing the autonomous and exploratory behaviors, as well as the level of anxiety, of animals in new settings. When combined with a depression model, it can be used to detect “depressive‐like” behaviors in animals and to evaluate the antidepressant efficacy of pharmacological treatments. The detection indicator is the animal's open‐field activity ability. The number of times the animal is modified and vertically standing is manually counted as the behavioral indicator. Simultaneously, automated cameras are utilized to capture the animal's movement trajectory, and behavioral analysis software is employed to analyze behavioral indicators including total movement distance, average movement speed, and duration of rest. The pentobarbital sodium righting reflex experiment reflex assay refers to the reflex that occurs when an animal is in an abnormal position after anesthesia and returns to a normal position (Kim et al., 2022). By recording the time at which the righting reflex was lost following pentobarbital sodium anesthesia in various groups of rats, the mental status of the different rat groups could be ascertained.

Therefore, this study used open‐field experiments and pentobarbital sodium righting reflex experiments as the foundational methods for behavioral detection. After analyzing the data from the open field experiment, it was found that compared to the control group and the treatment group, the model group and NR exhibited reductions in total distance traveled, average speed, number of standing instances, and number of rearing episodes, while the duration of rest increased. It can be inferred that the activity level of the model group and NR rats decreased after entering a new environment, indicating a decrease in their independent exploratory behavior and interest in the surrounding environment. However, the activity of the treatment group rats showed varying degrees of increase. The ability to explore independently and interest in the surrounding environment has also increased to a certain extent. After comparing and analyzing the disappearance time of the righting reflex in different groups of rats, it was found that the sleep latency of the model group was significantly increased compared to the control, indicating the success of the modeling. Compared with NR, the sleep latency of PC and MD significantly decreased, and the sleep time significantly increased. Although the changes in sleep latency and sleep time in HD and LD were not significant, both groups showed a trend of reducing the sleep latency of rats and increasing sleep time. From this, it can be inferred that MS essential oil can shorten the sleep time of rats and increase their sleep time. The effect of medium‐concentration MS essential oil is superior. Building on this, histopathological examinations were carried out on the inferior colliculus of the rats to further assess its therapeutic effectiveness.

Hematoxylin–eosin (HE) staining is one of the commonly used staining methods in paraffin sectioning technology. Hematoxylin is an alkaline dye that primarily stains the chromatin in the nucleus and nucleic acids in the cytoplasm a purple‐blue color. Eosin, on the other hand, is an acidic dye, which mainly colors the components within the cytoplasm and the extracellular matrix a reddish hue. It is one of the main colorants in histopathology and the most widely used colorant in medical diagnosis (Liu et al., 2017). Insomnia can cause damage to neurons and glial cells in the brain (Carvalhas‐Almeida et al., 2023). A study utilizing transgenic rats experiencing acute microglial depletion demonstrated that the biological clock genes and their corresponding proteins were disrupted, highlighting the role of microglia in governing the regulation of circadian rhythms (Sominsky et al., 2021). The morphological changes of microglia are believed to be influenced by circadian rhythms, and insufficient sleep can induce alterations in these cells (Hayashi et al., 2013). After the analysis of H&E staining results in this study, the model group and NR rats' nerve cells had many hyperchromatic nuclei, light cell staining, and small focal bleeding, indicating that the circadian rhythm of the two rats was severely damaged, further confirming the success of modeling. PC, HD, MD, and LD showed varying degrees of reduction in neuronal cell nuclear fixation and deep staining compared to NR. Cell staining returned to normal, with occasional bleeding but smaller bleeding points compared to NR. Prove that MS essential oil can to some extent improve and repair nerve cell damage caused by insomnia in the hypothalamus of rats. Combined with the results of open field experiments and sodium barbiturate righting experiments, it was further demonstrated that MS essential oil has the effect of improving sleep. Finally, factors related to insomnia in rats were detected.

The γ‐aminobutyric acid (GABA) and 5‐hydroxytryptamine (5‐HT) systems are pivotal in regulating the emotional circuits that underpin anxiety and depression, which are closely associated with insomnia (Hao et al., 2024; Lechin et al., 2004; Romana et al., 2020). According to reports, therapeutic agents exert their sedative and sleep‐improving effects by modulating the transmission of 5‐HT and GABA in the central nervous system (Cho et al., 2010). 5‐HT, also known as serotonin, plays a crucial role in sleep‐wakefulness regulation (Semyanov et al., 2004). The abnormality of 5‐HT signaling has been extensively documented in neuropsychiatric diseases, including depression, anxiety, schizophrenia, and autism spectrum disorder (Lesch & Waider, 2012). This experiment used the PCPA insomnia model because PCPA is a tryptophan hydroxylase inhibitor that depletes 5‐HT levels, thereby causing insomnia. GABA is a major inhibitory neurotransmitter in the central nervous system. The GABA receptor system plays a major inhibitory role in the brain and is essential in maintaining the overall balance between neuronal excitation and inhibition (Semyanov et al., 2004). Therefore, detecting GABA levels in the hypothalamus of rats is crucial for analyzing the efficacy of MS essential oil. The detection results from the ELISA kit revealed that following modeling, the serum 5‐HT and hypothalamic GABA concentrations in the model group rats were significantly reduced compared to those in the control group rats, confirming the successful establishment of the model. Following administration, there was a significant increase in GABA levels in the hypothalamus of the MD and LD groups. This suggests that MS essential oil has an efficacy in improving sleep. Additionally, the serum 5‐HT levels in the NR group of rats showed an abnormally high increase compared to the model group. Through reviewing the relevant literature, Heyan's analysis of the correlation between serotonin (5‐HT) levels and heart rate variability in generalized anxiety disorder among young patients noted that blood 5‐HT levels are closely associated with generalized anxiety. In Heyan's research, adolescents with generalized anxiety had higher levels of 5‐HT in their blood compared to healthy adolescents (Yan et al., 2015). Rachel Dott also mentioned in his work on the regulation of circadian rhythm in depression that circadian rhythm disruption may exacerbate depressive symptoms by influencing emotion‐related brain regions through the action of 5‐HT (serotonin) (Daut & Fonken, 2019). Based on the above literature, it is speculated that the abnormal levels of 5‐HT in NR of rats are higher than those in the control group and treatment group due to the disruption of the sleep cycle, which leads to depressive symptoms in the rats. From the side, it also reflects that MS essential oil has a therapeutic effect on insomnia.

Collectively, the underlying mechanism for the effects of combined inhaled aromatherapy on brain function involves the activation of nasal olfactory chemoreceptors and the subsequent olfactory signaling pathway (Cha et al., 2021). According to neurobiological studies, the olfactory nerve, which connects the olfactory system to the central nervous system, facilitates the processing of odor information (Schneider et al., 2019). Essential oil molecules via vapor inhalation enter the blood circulation through the alveoli of the respiratory system, and subsequently, small lipophilic molecules can easily cross the blood–brain barrier to affect the brain (Faturi et al., 2010). It was initially hypothesized that MS essential oil, once transformed into a steam jet by an ultrasonic aerosolizing machine, is inhaled by the rats into the alveoli of the respiratory system, where it enters the bloodstream. Subsequently, the small lipophilic molecules within the oil cross the blood–brain barrier, thereby influencing the brain. This process is thought to affect the levels of 5‐HT and GABA in the rat brain, thereby regulating sleep in the rats.

## 5 CONCLUSION

The main components of MS essential oil are oleic acid, palmitoleic acid, stigmasterol, etc. It can improve anxiety and tension in rats and has sedative and hypnotic effect.

## AUTHOR CONTRIBUTIONS

**Shaofeng Wei:** Conceptualization (lead); data curation (lead); funding acquisition (lead); investigation (lead); methodology (lead); project administration (lead); resources (equal); supervision (equal); validation (equal); visualization (lead); writing – review and editing (lead). **Ruijie Chen:** Formal analysis (equal); funding acquisition (equal); investigation (equal); methodology (equal); project administration (equal); resources (lead); software (lead); supervision (lead); validation (equal); visualization (equal); writing – original draft (lead). **Xiaoyi Liu:** Data curation (equal); methodology (equal); software (equal); supervision (equal); validation (equal); visualization (equal); writing – review and editing (equal). **Haoran Ma:** Formal analysis (equal); investigation (equal); methodology (equal); supervision (equal). **Yang Peng:** Software (equal); supervision (equal). **Xiefei Wu:** Software (equal); supervision (equal). **Yong An:** Software (equal); supervision (equal). **Xinru Wang:** Software (equal); supervision (equal). **Peng Luo:** Data curation (lead); formal analysis (lead); funding acquisition (lead).

## FUNDING INFORMATION

This research was conducted being taken the support of the Guizhou Science and Technology Department Plan Project (Guizhou Science and Technology Foundation‐ZK [2023] General 315), the Guizhou Science and Technology Combined Support [2021]134, Innovation and Entrepreneurship Talent Project of Guizhou (number S202210660122), and the Guizhou Province's first‐class discipline construction project, Public Health and Preventive Medicine (number 2017).

## CONFLICT OF INTEREST STATEMENT

The authors declare that the research was conducted without any commercial or financial relationships that could be construed as a potential conflict of interest.

## Supporting information


Appendix S1.

## Data Availability

The original contributions presented in this study are included in the article/supplementary material; further inquiries can be directed to the corresponding authors.
