# Supplementary figures and images for: Aromatherapy was used to explore the sedative and hypnotic effects of Moringa seed essential oil on insomnia rats
Source: Food Sci Nutr. 2024 Nov 12;12(12):10463–76. doi: 10.1002/fsn3.4484 (PMC11666963; doi:10.1002/fsn3.4484)

**Appendix**

Appendix1：


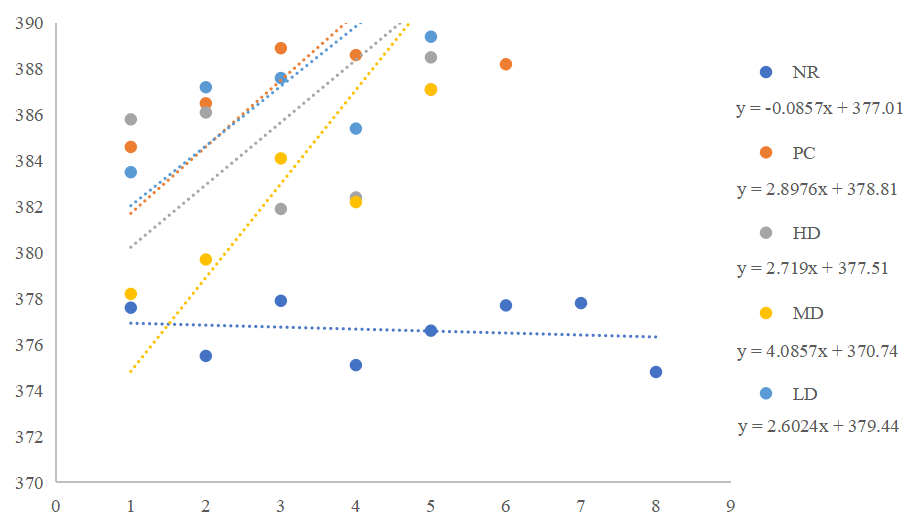


Fig. S1 Weight change trend chart

Supplement: Supplementary file 1 — Appendix S1. [file FSN3-12-10463-s001.docx]
